# Social sensing of floods in the UK

**DOI:** 10.1371/journal.pone.0189327

**Published:** 2018-01-31

**Authors:** Rudy Arthur, Chris A. Boulton, Humphrey Shotton, Hywel T. P. Williams

**Affiliations:** 1 Computer Science, CEMPS, University of Exeter, Laver Building, North Park Road, Exeter, EX4 4QE, United Kingdom; 2 Earth System Science, CLES, University of Exeter, Laver Building, North Park Road, Exeter, EX4 4QE, United Kingdom; Bristol University/Remote Sensing Solutions Inc., UNITED STATES

## Abstract

“Social sensing” is a form of crowd-sourcing that involves systematic analysis of digital communications to detect real-world events. Here we consider the use of social sensing for observing natural hazards. In particular, we present a case study that uses data from a popular social media platform (Twitter) to detect and locate flood events in the UK. In order to improve data quality we apply a number of filters (timezone, simple text filters and a naive Bayes ‘relevance’ filter) to the data. We then use place names in the user profile and message text to infer the location of the tweets. These two steps remove most of the irrelevant tweets and yield orders of magnitude more located tweets than we have by relying on geo-tagged data. We demonstrate that high resolution social sensing of floods is feasible and we can produce high-quality historical and real-time maps of floods using Twitter.

## Introduction

Natural hazards such as floods, wildfires, storms and other extreme weather events cause substantial disruption to human activity and are predicted to increase in frequency and severity as the climate changes [1, 2]. Rapid high-resolution observations of unfolding hazard events can inform decision-making and management responses. However observations from meteorological and climatological instrumentation typically suffer from data sparsity, time delays and high costs [2]. Furthermore, the human impacts associated with natural hazards are hard to measure using sensor platforms designed to observe the hazards themselves. Rain gauges and stream gauges can only measure the amount of precipitation, or the height of floodwater, not the impact on people’s lives. Crowd-sourcing has been proposed as a possible solution to the need for better observations of environmental phenomena and associated impacts, utilising a variety of methods including citizen observations, web technologies, distributed sensor networks, and smart devices [2].

A useful distinction may be drawn between “solicited” crowd-sourcing (where users are somehow recruited to participate in structured observation programmes) and “unsolicited” crowdsourcing (where observations are derived as a by-product of social communication for other purposes). Most citizen science and crowdsourcing studies to date have generated solicited observations [2]. Examples relevant to natural hazards include the UK Met Office “Weather Observation Website” [3], which allows amateur meteorologists to upload local weather reports through a web application, the European Severe Weather Database [4] which uses eye-witness reports to map severe weather across Europe, and the UKSnowMap [5] initiative that uses a particular hashtag to allow users to contribute structured snowfall observations via social media. Solicited crowd-sourcing tools typically use a designed interface to impose structure on the data that is collected, which may improve data quality by standardising the citizen observations obtained. However, such tools rely on dedicated volunteers choosing to upload data and the volume of observations that is collected may therefore be limited.

Social sensing is here defined as observation of real-world events using unsolicited content from digital communications (e.g. mobile phone call records, social media, web searches, and other online data). The core challenge of social sensing is to extract high-quality observational data from large numbers of unstructured, patchy, and possibly inaccurate user utterances, or from associated metadata. Call data records (CDR) from mobile phone usage have been analysed to reveal patterns of mobility in response to natural disasters [6] and to predict the spread of infectious diseases [7, 8]. However, CDR data is hard to access due to potential commercial sensitivity and privacy concerns. Web search statistics have also been used to track infectious diseases, but are affected by changes in underlying (proprietary) search algorithms which may change outputs and confound statistical analyses [9]. In this study we consider social sensing using publicly available social media data.

Several studies have used social media for sensing natural hazards. Data from Twitter has been used for detection/location of earthquakes [10], typhoons [10], wildfires [11] and heat waves [12]. Data from other platforms has also been used, including air quality prediction in China using data from Sina Weibo [13] and flood prediction using Flickr [14].

The early work of Sakaki et. al. [10] proposed a classifier based on keywords in a tweet, the number of words and their context to produce a probabilistic model for earthquakes that located their centre and predicted the trajectory of the shock. They managed to predict 96% of significant earthquakes in Japan and moreover were able to produce warnings faster than the Japan Meteorological Agency. This methodology also worked well for typhoons and demonstrated that information on Twitter could be of immense benefit for studying natural hazards. Boulton et. al. [11] showed that Twitter could be used to detect wildfires in the US, with tweets about fires showing strong spatio-temporal correlation with satellite data. Similarly Kirilenko et. al. [12] showed that Twitter users identify heat waves. The work of Sakaki et. al. was influential, however most studies using Twitter to detect events have focused on epidemics e.g. [15] or political events [16] rather than natural hazards *per se*. The field of natural hazard monitoring using Twitter remains fairly under-studied, and certainly many meteorological and disaster management agencies who could benefit from this data are not currently using it.

The advantage of social media data is volume. For example, the popular social media platform Twitter has over 320 million active users each month producing over 500 million tweets each day [17]. The accessibility and real-time information dissemination capabilities of Twitter make it a good candidate for social sensing in the geographical areas where it has high usage. However, Twitter has an important limitation in that only a small fraction (typically <1%) of tweets have accurate location information in the form of latitude/longitude coordinates derived from a GPS-capable mobile device. Instagram has a higher proportion of GPS-tagged content (e.g. ∼12% of posts were geotagged in a social sensing study of wildfires in the USA [11]). Facebook has very high usage but privacy constraints severely restrict its use for social sensing.

A general problem for crowd-sourcing of environmental observations is data quality. The potential for high-volume and high-resolution observations is balanced against the potential for inconsistent, inaccurate and subjective reporting. Social media content suffers from a lack of structure and standardisation in the observations that are collected, and “noise” in the form of irrelevant content returned by search filters. Social media attention to “routine” daily weather may be low, with more attention given to “newsworthy” extreme events. The networked nature of social media may exacerbate these problems, since users are exposed to each other’s content and are likely to have a relatively high density of online connections to other users from the same geographical region [18], the independence of observations arising from a particular region is unknown. Interpersonal influence and social spreading of content (often mediated by mechanisms designed for this exact purpose, for example, retweets, re-posts, likes) may amplify or suppress some kinds of user report and thereby distort observations.

Here we describe a case study of social sensing of flooding in the UK using unsolicited data from Twitter. Since the geographical location of events is important for this application, we use location inference to estimate the geographical origin of tweets that do not contain GPS coordinates, adapting a method [19] that utilises a variety of indicators taken from the tweet object and user profile to infer the location of the user at the time the tweet was written.

This paper has several aims. The first is to discover if social sensing can be used to detect flood events as has been demonstrated for other hazards, such as earthquakes and wildfires. Once this has been shown we want to use Twitter to produce data for flood forecasters in order to help validate and improve their forecasts, particularly of minor floods. At present the methods to validate flood forecasts rely on manual searching of national and local news in areas where there has been a flood forecast. It would be useful to forecasters firstly to have an automated method which removed the burden of performing this manual search and secondly to have a method which can detect very localised floods, which may not make the news. Finally, we note that the tweets we collect often contain useful information about transport disruption, the progress of the flood waters and emergency service response. Thus by spatially locating all this information we can provide historical and real-time maps which could be of use to emergency responders, planners, insurance agencies and others interested in measuring the human impact of floods.

In Section 1 we first describe our method of data collection as well as our validation dataset. Section 1.1 describes how the data was collected, Section 1.2 describes how we filter the data stream for relevant tweets in the right geographical area. Section 1.3 describes our approach to location inference and 1.4 shows how we detect flood events given relevant, located tweets. Section 2 covers the outputs of social sensing, validation and parameter tuning. We also show an example of a day with an extreme flood event as an example of how social sensing could be used in practice. Section 3 offers some simple conclusions and discusses the potential/limitations of social sensing.

## 1 Methods

The overall method has four stages: data collection, content filtering, location inference and event detection. These are covered in turn below. Choices are made at each stage which affect the final outcome of the analysis. We use the performance of the overall system to tune parameters for the location inference and event detection methods.

### 1.1 Data collection

#### 1.1.1 Twitter dataset

**Fig 1:**
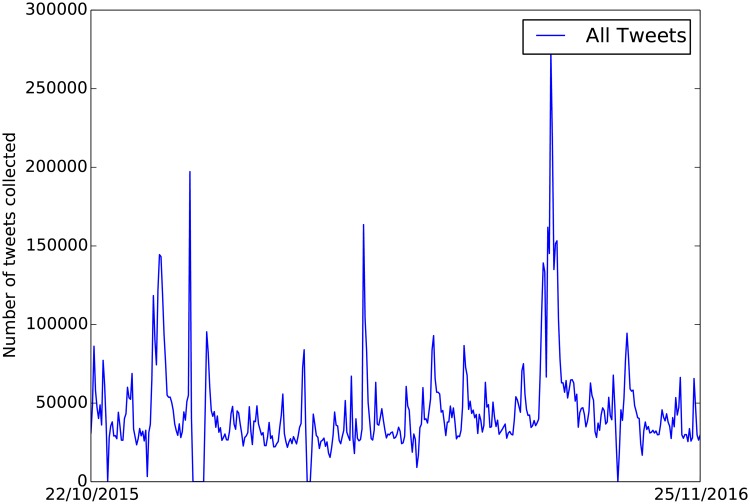
Number of tweets collected per day during the whole collection period 22/10/2015 and 25/11/2016.

Tweets were collected from the Twitter Streaming API using the search terms “*flood, flooding, flooded*”. All data was collected according to Twitter’s terms of service and privacy conditions. Collection scripts were implemented in Python using the Twython [20] package. Twitter returns a JSON object for each tweet, this is a common data exchange format consisting of a collection of key-value pairs. The JSON object contains the tweet content and various meta-data (time stamp, geotag, user profile, etc.). A timeseries of daily tweet counts for this period is shown in Fig 1.

#### 1.1.2 Validation dataset

We validate our social sensing method against a database of flood observations collected by the Flood Forecasting Centre. These data record the date, location (resolved to the level of the UK administrative area in which the flood occurred) and severity of floods (severe/major/minor) over a period from 01/01/2015 to 23/11/2016. These data are obtained by manually searching local and national news for reports of floods and using alternative social media monitoring tools. This data represents a necessarily incomplete picture of floods happening in the UK over this period; data are only compiled when floods have been forecast. There are likely to be few or no “false positives” where floods were recorded but never occurred, but an unknown number of “false negatives” where floods occurred but were not recorded. We will take these considerations into account when it comes to tuning the parameters of our algorithm.

### 1.2 Filters

A top-level filter is instantiated by the search string passed to the Twitter API (see above). While it is possible to constrain the search at the level of the API, here we prefer to use a general API call and filter the returned content post-collection. We use several additional filters to remove undesirable content, applied in the order below.

#### Timezone filter

The user timezone has been used elsewhere as an indicator for geolocating tweets [19]. For this study the target area is decided beforehand and tweets by users with timezones set outside this area are discarded. This has the potential to erroneously remove some content from users who have unset, or incorrectly set, timezones. However, checking for wrongly set timezones requires a lot of processing and would make real-time analysis difficult. Here we require that users have their timezone set to one of *London, Edinburgh, UTC*.

#### Bot filter

Some Twitter accounts are automated “bots” that tweet a high volume of flood-related messages. These can often be identified by their anomalously high level of activity, operationalised here as accounts whose activity makes up more than ∼1% of the total tweet volume. We found three such users *@RiverLevelsUK, @UKFloodTweets, @FloodAlerts*. Since we are interested in *social* sensing we discard tweets from these users.

#### Retweet filter

Many tweets are users quoting or relaying each other’s messages. We are interested in original and direct flood observations by individuals (e.g. *“Can’t believe my garden is flooded”*) or organisations (e.g. *“We are closed tonight due to flooding”*), rather than acknowledgements or forwarding of these observations by others. Thus we filter out retweets.

#### Relevance filter

Twitter users often use the search keywords used to collect data (*flood, flooding, flooded*) in contexts that are unrelated to our topic (e.g. “*The market was flooded with copies*.”). To remove irrelevant content, we first remove tweets containing some manually curated filter terms,

“flood with” “flood of” “flood in” “flood it” “flooded with” “flooded by” “flooding back” “immigrant” “migrant” “migration” “market” “tears” “flood-hit”

We then apply a machine learning relevance classifier to the remainder. To develop the classifier, we first observed that content typically showed four identifiable classes of tweets about floods (with some tweets remaining ambiguous):

- **Irrelevant** e.g. *I was in floods of tears*
- **Historical** e.g. *Charity raffle for Cumbrian flood victims*
- **Warnings** e.g. *River Ouse water level: 2.14m. Chance of flooding*.
- **Immediate** e.g. *It is flooded outside*.

For forecast verification and flood tracking we want to focus on the **Immediate** class: these are the useful social data for detecting floods currently happening in the UK. We manually curated a set of 3879 tweets, classifying each tweet as **Immediate** or **Other** (including the Irrelevant, Historical and Warnings categories identified above) and used them to train a Naive Bayes [21] classifier. These tweets were randomly chosen from tweets which passed the other filters and manually classified. To build the classifier we first vectorized the data by counting single-word and 2-gram (two-word) occurrences in the training corpus. We used a multinomial naive Bayes classifier [22] with smoothing parameter *α* = 0.5, the exact value of *α* is not crucial, the results below are very similar for a range of possible smoothing parameter values. To validate, we first split the training data and find 80% of tweets classified correctly when the model is built from 75% of the data. Then, using all the data, we perform 6-fold cross validation. We find the sum of the confusion matrices from each of the 6 cross validations is

$$\begin{array}{c} \left(\begin{array}{cc} TN & FP\\ FN & TP \end{array})=(\begin{array}{cc} 1759 & 359\\ 337 & 1360 \end{array}\right) \end{array}$$

These results imply that the classifier is not biased towards false negatives or false positives.

### 1.3 Location inference

As stated above, typically <1% tweets have GPS coordinates attached as metadata. Location inference is therefore an active topic in analysis of social media. Our method is based on the multi-indicator approach developed by [19], which uses a variety of indicators to infer tweet origin, including user timezone, place names mentioned in tweet text, user location field, GPS coordinates, etc. However, unlike other studies using location inference [19, 23–25], here we are not trying to locate individual users and tweets, but to locate flood events. Our variant of the location inference problem allows for some simplification compared to the general problem of locating a given tweet without any contextual information.

In order to provide for potential usage for real-time updates, the location inference process must be sufficiently fast. As we will describe below, calls to some external databases are needed to identify place names (toponyms) in tweets. The public interfaces of these services are rate-limited and, since they require user accounts, we cannot perform multiple queries in parallel. DBPedia Spotlight implements an algorithm to detect toponyms, which can take time (seconds) to yield results. These facts can be an issue during active periods, e.g. a large flood in a populous area. This means it is important to only try to locate relevant tweets, rather than every tweet that is collected, so the filtering described above is of key importance. Since we suppose that the algorithm developed here will be applied to detect floods in a specific area (e.g. our study focuses on floods in the UK) we utilise the user timezone associated with each tweet as a simple filter prior to location inference, rather than as part of a generalised location inference procedure.

If a tweet passes through all the filters it is likely (though not certain) to be relevant, i.e. to concern a flood currently happening somewhere in the UK. The two most important indicators for locating the tweet more precisely (assuming the absence of GPS coordinates in metadata) are place names mentioned in the tweet text and place names mentioned in the user location field. Since the flood-focused tweets analysed here are relatively likely to mention specific places (i.e. where the flooding is occurring) in comparison to other studies [19, 25], and since we are seeking to locate floods rather than user home locations, we can assume that message text is more likely to contain useful location information than the user location metadata. For example the tweet text “*Train from Exeter to Totnes cancelled due to flooding on the track*.” is very useful for location inference and is more relevant than the location field for the train company which authored the tweet, which contains “South-West UK”. This fact will be reflected in the relative weighting of text and location fields in the inference process. Since we are ultimately trying to locate floods (not people or tweets), when tuning parameters and other design options, we focus on the overall accuracy of flood event detection, rather than the accuracy of location inference on a single tweet, as the metric for improvement.

#### 1.3.1 Method

A small subset of tweets have a *geotag*—precise GPS co-ordinates (from a GPS unit in a mobile device) of the tweet’s origin included in the metadata of the tweet object. For the small subset of geotagged tweets we can simply use the GPS co-ordinates and no inference is necessary. For the majority of tweets, which are not geo-tagged, we use the method below, based on location information from the user location field and the tweet text. To look up polygons outlining named areas we make use of GADM [26], a spatial database of the location of the world’s administrative areas or administrative boundaries.

**Location Field**: A small number of users have GPS co-ordinates in their location field (these are independent of any GPS information in tweet metadata), usually automatically added by a mobile device. For those users we use this GPS co-ordinate. The vast majority of users have text in their location field which we look up in the online gazetteer Geonames [27], which, if successful, returns possible locations and a similarity score, indicating confidence in the result. If no result is found by looking up the whole location field we split the text by commas, slashes or hyphens and look up each entity separately. Geonames provides the country each location is in. Since we are concerned with floods in the UK we keep only results from the UK, disambiguating e.g. Boston, Linconshire from Boston, Massachusetts but not Cambridge, Gloucestershire from Cambridge, Cambridgeshire. If Geonames reports the place as a ‘region’, ‘area’, ‘city’ or ‘state’ we look up the location in GADM to obtain a polygon describing the location, otherwise we use the latitude and longitude provided by Geonames. We store the polygons or coordinates, together with the similarity score, which we use as an approximate quality score for each possible location indicated by location field.

**Message Text**: We use DBPedia Spotlight [28] to identify all entities in the whole tweet message text and link them to DBPedia [29] entries. If DBPedia Spotlight tags an entity as a place, we first attempt to look up the place in GADM. If the place is not found in that database we follow the link to DBPedia and use the latitude and longitude obtained from there. DBPedia Spotlight returns a similarity score for each entity it tags, which we use as a quality score.

**Overall Inference**: The results of this process are several polygons or coordinate pairs with different quality scores. For example, for a tweet with a user location field containing *Cumbria / London* and the message *Terrible flooding in Carlisle today*, we would obtain three objects (two polygons for Cumbria and London, and point coordinates for Carlisle), each with a different quality score. To obtain the most likely origin of the tweet, taking into account the differing quality of each estimated location, we weight each polygon/point by its quality score and calculate the sum of the weights of intersecting areas. This example would yield Carlisle, which is in Cumbria, as the most likely location (i.e. the location with the highest summed weight). In this way we obtain a list of the most likely points or polygons associated with each tweet. It is also possible to skip this step and keep all the polygons associated with every tweet, but this was found to give slightly worse agreement with the verification data, so we use the overlap method and keep only the most likely location. As we mentioned before, the importance of place names mentioned in the text versus place names in the location field may be different. We multiply the weights of the message polygons by a factor *r*, where *r* will be tuned to optimize agreement with the verification data.

### 1.4 Flood event detection

After filtering and location inference, we are left with a set of relevant geo-located tweets which we take as local observations of floods. The next task is to combine these tweets into a temporally resolved map of flood events for comparison with our validation dataset from FFC.

We add all tweets collected over some period, usually 24 hours, to a map to find the places with the highest volume of flood related tweets. To this end we start with a spatial bounding box containing England and Wales (Scotland and Northern Ireland are not included in our verification dataset) and divide it into an *N* × *M* grid. Every grid square, *g*, initially has height *g*_*h*_ = 0. For every polygon *p* and grid square *g* we add a value proportional to their overlap:

$$\begin{array}{c} g_{h}\to g_{h}+\frac{\text{Area}\;\text{of}\;g\cap p}{\text{Area}\;\text{of}\;p} \end{array}$$

When *p* is a point location, or entirely inside a grid square, we add 1 to the grid square containing *p*. This means every tweet counts equally, but if a tweet is only roughly located e.g. *p* is a polygon covering all of Wales, we will add a very small amount to each grid square intersecting Wales. This process privileges precise locations, meaning that tweets for which GPS coordinates are available will contribute strongly to flood detection.

Population density must be accounted for since, for example, the total volume of tweets from London is so large that, based on absolute numbers, it almost always appears flooded. Ideally we would use the total number of tweets from an area to normalise, however we do not have access to this data. We use the 2011 UK census [30] to obtain very fine grained (though slightly out of date) population density information which allows us to calculate the population inside each grid square, *N*_*g*_. We then scale the heights

$$\begin{array}{c} g_{h}\to \frac{g_{h}}{N_{g}^{\alpha }} \end{array}$$

where *α* is an adjustable parameter. *α* = 0 removes the scaling for population density, while increasing *α* more severely down-weights grid squares with large populations and hence gives more importance to tweets about floods from sparsely populated areas. We find appropriate values for *α* by tuning its value based on overall flood detection performance (see below).

## 2 Results

### 2.1 Data preparation

**Fig 2:**
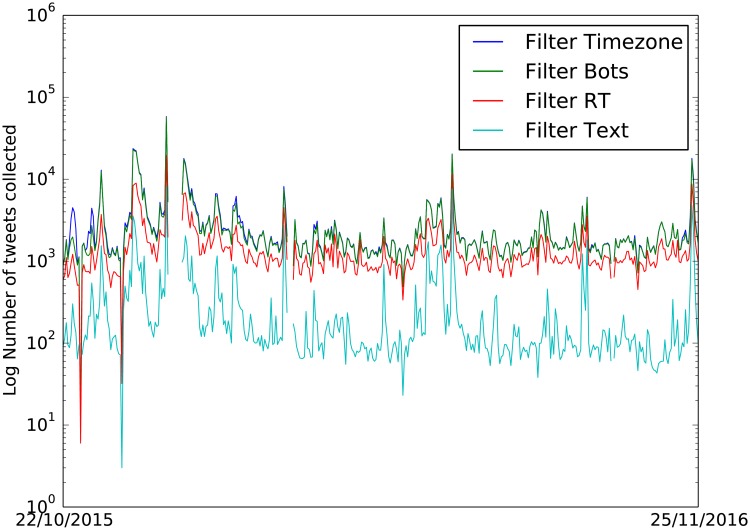
Number of tweets collected per day during the whole collection period 22/12/2015 and 04/01/2016 at each filter level.

**Fig 3:**
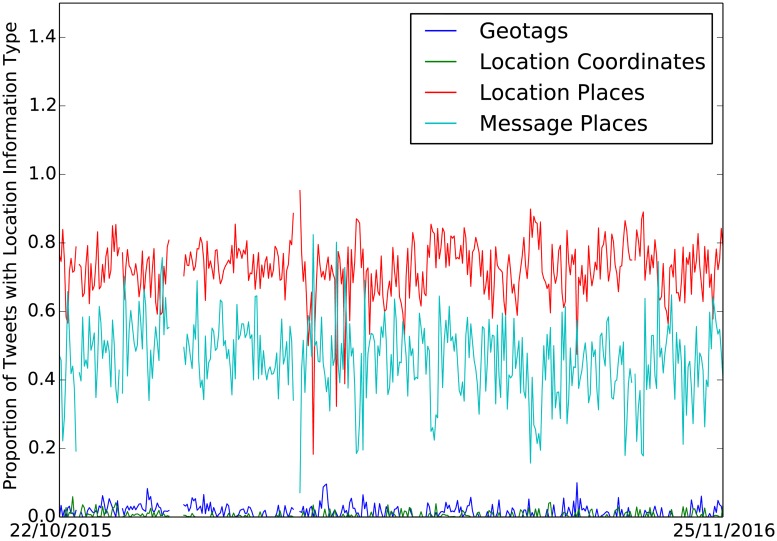
Number of relevant tweets collected with location info in each field: GPS-tagged tweets, location field GPS coordinates, location field toponyms, message text toponyms.

17,828,704 tweets were collected between 22/10/2015 and 25/11/2016 with a gap in collection between 28/12/2015 and 04/01/2016 inclusive. Table 1 shows the numbers of tweets retained after each filtering step. Figs 2 and 3 show daily tweet counts broken down by filtering and location inference outputs. In order to get a rough idea of the benefit of each filtering step we measure the Pearson correlation between daily tweet counts and the FFC validation data. To make the FFC data more directly comparable, we create a count for each day by summing the populations of the flooded counties multiplied by the severity of the flood in the county (using a multiplication factor of 1 for a minor flood, 2 for a significant flood and 3 for a severe flood). This construction assumes that the number of tweets is directly proportional to population and that more severe floods generate more tweets. The result of this comparison is shown in the last row of Table 1. Each of the filters (apart from bot removal which is necessary to remove redundant forecast information) increases the correlation. This general pattern is robust to different transformations of the FFC data, though the amount of correlation changes. See Table 2 for details of location information associated with the filtered tweets. Of the 122,281 tweets retained after filtering, 79,163 had some form of location information associated with them, with just 1,574 having a geotag.

**Table 1:** Total number of tweets remaining after each filter is applied and correlation of the number of tweets per day with FFC data.

	All	Timezone	Bot	Retweet	Relevance
Tweets Remaining	17828704	1105360	1051295	582141	122281
Correlation	0.206	0.551	0.550	0.591	0.673

**Table 2:** Total number of tweets with each kind of location information.

All Tweets	Any Location Info	Geotag	Loc: GPS	Loc: Toponym	Text: Toponym
122281	79163	1574	349	59179	41315

Tweets can have location information in multiple fields: the Geotag, the location field (Loc), or in the tweet message itself (Text).

### 2.2 Real time flood maps

**Fig 4:**
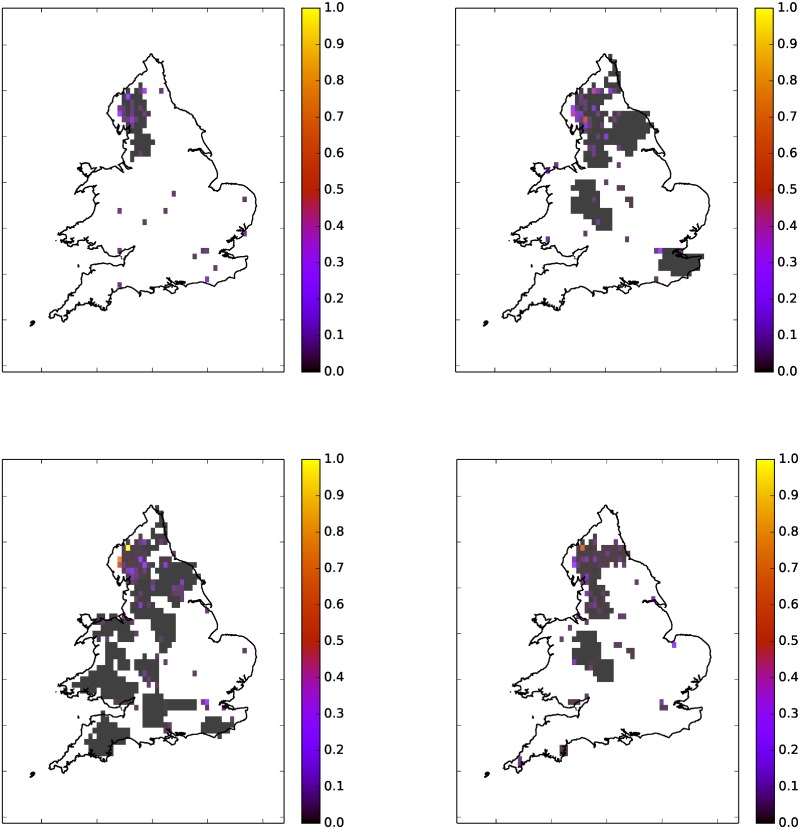
Floodiness grid, 64 × 64, over England and Wales on 5/12/2015 using (*r*, *α*, *T*) = (1.0, 0.15, 0.1). Using tweets collected in 1 hour windows. White indicates no tweets. Colour bar units are floodiness relative to daily max. Top left: 10am-11am. Top Right: 1pm-2pm. Bottom Left: 4pm-5pm. Bottom Right: 9pm-10pm.

During large floods there is likely to be a high volume of Twitter activity referencing the event. Thus we can make ‘live’ maps of Twitter activity which may be of use to individual travellers, train and bus companies (though these companies provide a lot of the most useful tweets) or flood management agencies. Fig 4 shows an example of four one hour periods on a particularly floody day: 05/12/2015 which saw severe flooding in the north of England.

**Fig 5:**
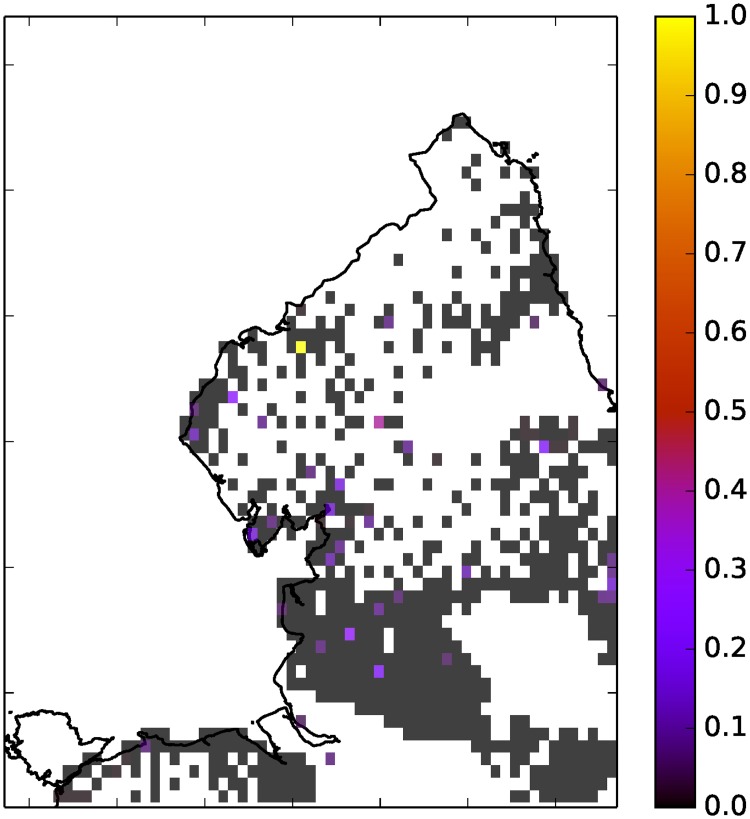
Floodiness grid, 64 × 64, over the North East on 5/12/2015 between 4pm and 5pm using (*r*, *α*, *T*) = (1.0, 0.15, 0.1). White indicates no tweets or zero population. Colour bar indicates floodiness relative to daily max.

In Fig 4 there are indications of floods early in the morning. Later in the afternoon we can see flood hotspots in three places, a town in the area (Kendal) and on the road between the two major cities in the area Carlisle and Newcastle. In the early evening there is a lot of activity in Carlisle, which continues into the night. If there are sufficiently many tweets the grid can be refined to provide more detailed local information, as in Fig 5.

### 2.3 Validation and tuning

To validate our method we use the manually curated flood observation dataset provided by FFC. Since our algorithm detects floods by the volume of tweets observed in arbitrary grid squares, whereas the FFC system uses UK administrative areas (typically counties) we need to map our grid data onto the same geographical regions used by FFC (hereafter counties). There are several ways to do this, from which we select a method that mimics how floods are recorded by the FFC. For each observation period (24 hours from midnight to midnight) we calculate the grid for some choice of parameters *r* (relative weighting of message text and user location field in location inference) and *α* (population density scaling). We divide by the maximum height so that the highest square has *g*_*h*_ = 1. We then set a threshold *T* and use the criteria: if a grid square overlapping a country has *g*_*h*_ greater than *T* we declare that county flooded and check for agreement with the FFC data.

If we call *g*_*h*_ ‘floodiness’ then by doing the normalization we are measuring relative rather than absolute floodiness. This means, even on a day with no floods anywhere in the UK, some particular point will be flagged as flooded. Both absolute and relative floodiness are useful metrics and both should be reported. One of the use cases of this method is for forecast verification and flood tracking. In this case the relative measure, which highlights the most flooded areas, will be useful, even if the day is not particularly ‘floody’. In other situations, e.g. fine days with no floods forecast, the absolute measure is more useful. Indeed, on days with no floods it is unnecessary to try to socially sense them! When we are trying to socially sense floods we will have a good reason to expect a flood somewhere—e.g. one was forecast, heavy rainfall.

For tuning we chose one day at random from each month where we had both verification data from the FFC and had collected tweets, since days where FFC do not observe floods do not provide the necessary data for validation. We need to span a long time period in order to capture possibly different patterns of twitter use e.g. floods in summer compared to floods in winter may cause different patterns of tweeting.

In the following a *positive* is a county where (for some values of *r*, *α*) the floodiness was larger than *T* in some grid square intersecting that county and a *negative* is a county intersecting no grid square with floodiness larger than *T*. True positives (TPs) then are counties socially sensed to be flooded and also recorded in the FFC data, false positives (FPs) are socially sensed to be flooded but not in the FFC data. False negatives (FNs) are counties marked by the FFC but not social sensing.

To tune we choose 14 days where we have both FFC observations and tweets and count the number of TPs, FPs and FNs for different triples of *r*, *α* and *T* on each day. We then calculate

$$\begin{array}{c} \text{Precision}\;=\frac{TP}{TP+FP}\;\text{Recall}\;=\frac{TP}{TP+FN} \end{array}$$

for each parameter set for each day and compute average precision and recall over the 14 days. Using average precision and recall in this way means that each day contributes equally to the final scores, whereas compiling all TP/FP/FN scores into a single confusion matrix for calculation of a single precision and recall value would risk over-fitting to a single day.

**Fig 6:**
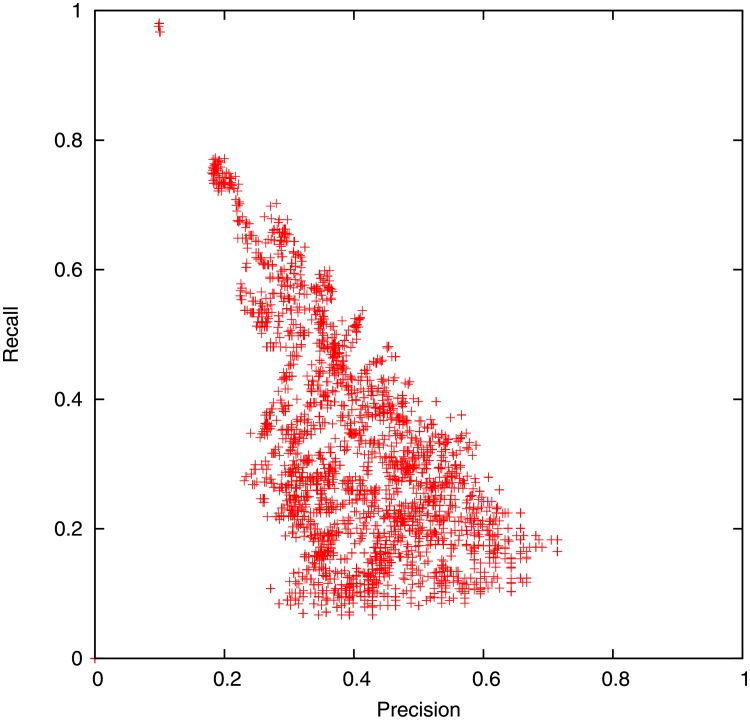
Tuning relative floodiness threshold *T* by varying text versus location weighting *r* and population scaling exponent α. Each point corresponds to the average precision and recall over 15 days for a different triple of *r*, *α*, *T*.

**Fig 7:**
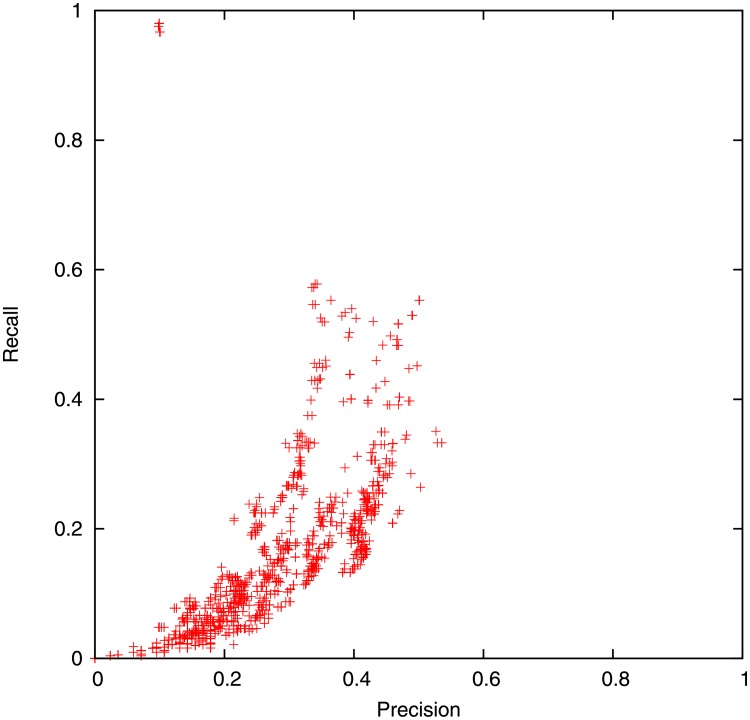
Tuning absolute floodiness threshold *T* by varying text versus location weighting *r* and population scaling exponent *α*. Each point corresponds to the average precision and recall over 15 days for a different triple of *r*, *α*, *T*.

Figs 6 and 7 shows the average precision and recall for different parameter sets. Recall may be much more important than precision i.e. it’s better to get a false alarm than to have no warning before a disaster. This is partly captured by the *F*_*β*_ score,

$$\begin{array}{c} F_{\beta }=(1+\beta^{2})\frac{\text{precision}\times \text{recall}}{\beta^{2}\text{precision}+\text{recall}} \end{array}$$ (1)

which is roughly a measure of accuracy given that recall is *β* times as important as precision. The precision and recall depend more sensitively on *α* and *T* than *r*. We only show the best parameter sets in Table 3, however many other triples of (*r*, *α*, *T*) are quite close or equivalent.

**Table 3:** Precision, recall and parameter set obtained by maximising *F*_*β*_ scores using absolute and normalised floodiness.

*β*	Max *F*_*β*_	Precision	Recall	(*r*, *α*, *T*)
**Relative**				
1	0.47	0.45	0.54	(≥ 2, 0.35, 0.25)
2	0.52	0.28	0.70	(≥ 1, 0.15, 0.1)
**Absolute**				
1	0.53	0.50	0.55	(≥ 2, 0.4, 0.075)
2	0.54	0.50	0.55	(≥ 2, 0.4, 0.075)

**Fig 8:**
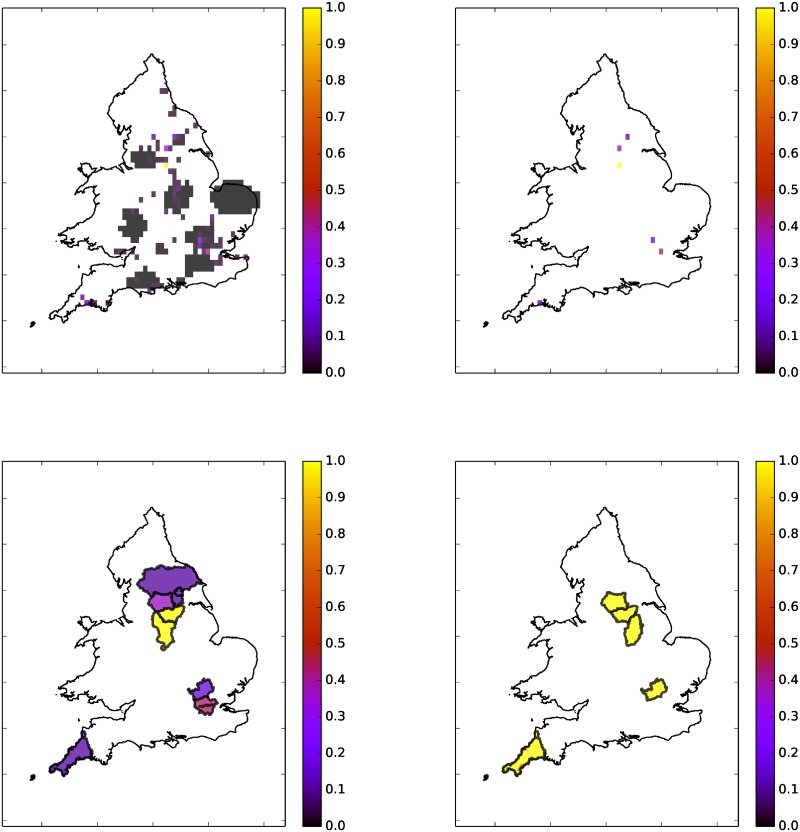
Flood map generated by twitter converted into FFC format for validation. White indicated no tweets. Colour bar units are relative floodiness. Top Left: Floodiness grid (64 × 64) over England and Wales on 28/10/2015 using (*r*, *α*) = (1.0, 0.15). Top Right: Showing only grid squares above threshold 0.1. Bottom Left: Counties with floods on 28/10/2015 according to Twitter. Bottom Right: Counties with floods on 28/10/2015 according to the FFC, with *g_h_* set to 1 for flooded counties.

Fig 8 shows an example of the grid constructed from one day, 28/10/2015, of flood tweets for one parameter set. We collected 48886 tweets in total for this day, of which only 302 passed all of the filters. Of these only 7 were geotagged while 222 had some form of location information in either the message text or the location field. There is reasonable agreement here at the level of counties, however Fig 8 clearly shows that the twitter data is much higher resolution than county level. Counties have very variable sizes and populations, and the population within a county is likely to be heavily concentrated in cities and towns—which are the places where floods generate impacts that can be socially sensed most easily. The higher resolution grid is more useful and more accurate and we only transform it into a map of counties for verification purposes.

Though likely to be more accurate than Twitter data, the FFC data we have is also not complete. Inspection suggests that many of our apparent false positives, using the FFC data as truth, represent real flood events. e.g.

‘Tidal #Flooding @WarehamDorset this morning’

‘M20 J2 London bound entry slip is closed due to flooding’

‘@LeicsCountyHall, you need a highways team out urgently to Clarence St., Loughborough as gullies on both side are blocked and road is flooded’

are all missing from the FFC data but seem likely to be reports of real floods. This suggests that our estimates of recall and especially precision may be too low and social sensing may be more accurate than suggested by comparison with FFC data. We suggest that the parameters *α*, *T* be changeable at the user’s discretion. The parameter *r* is difficult to change (requiring recomputing all the polygon overlaps) and but does not affect precision and recall as much as *α* and *T*, therefore we suggest fixing it at some *r* ≥ 1. Lower *T* improves recall but generates more false alarms, higher *α* emphasises rural floods more than cities.

By showing that very high recall is possible we have demonstrated that we can reproduce the validation data using Twitter alone. The lower precision indicates some false positives but, by manual examination of the Tweets, shows us that we are detecting a lot of real floods not present in the validation dataset. Thus the socially sensed flood map provides a more complete record of historical floods than the FFC data. For verifying forecasts, showing every flood that occurred may be desirable. However for other uses—emergency response, detecting transport disruption, calculating insurance premiums—floods occurring in populous areas are of greatest interest and the threshold should be chosen differently. Thus we show the results for a range of *α*, *T* values and suggest that the end user find the best parameter set for their own purposes.

We also note that many false positives are references to historical floods e.g.

‘Flood-damaged Leeds museum to re-open’

which were not caught by our text filters. Tweets mentioning historical floods may be of interest in analysing flood impact—more impactful floods will have longer ‘tails’ as people continue to talk about their effects. This is the subject of ongoing work.

## 3 Discussion

We have found that social sensing of floods is possible. The very low volume of geo-tagged tweets makes location inference a necessity and the accuracy of the location inference is crucial for the accuracy of the flood detection. We have outlined one location inference method here, which closely follows [19], though we added some filtering steps to quickly localise tweets in the target area. We could imagine more elaborate systems e.g. where a user’s friends contribute to their localisation [25] or where users who consistently contribute accurate information are weighted more highly. We are actively pursuing these paths. Furthermore other major events will likely generate significant discussion on social media e.g. extreme wind, heat or cold; storms and hurricanes; earthquakes and very similar methods could be applied to all these cases.

This approach does have biases, for example, we have more Tweets from areas with higher population density. We try to correct for this using the *α* parameter, but there is also likely to be a demographic bias, since the Twitter user-base is not representative of the population in general. Other factors may also affect the volume of tweets collected e.g. the time of year (Christmas) or time of day (8am on a weekday) of the flood peak; how surprising the the flood is or if the flood is covered by national news. These biases are an issue for forecast verification and our relevance filter and population scaling attempt to correct for them. From the point of view of ‘live’ flood tracking however, Twitter data provides low cost, and highly relevant information—since people only tweet about things which are of interest!

We are currently working with the FFC to use social media data to improve their validation datasets and replace the current manual process of scouring local and national news for flood coverage. This should give flood forecasters richer and more reliable verification data to work with when improving their predictions. We are also working on a web based version of this code which is easier to interact with, with the aim of presenting it to emergency services, train and bus companies, councils or any other organization interested in assessing the historical impact of floods. A real time version, accessible on the web, would also be of interest e.g. for individuals worried about transport disruption. For both of these projects we must be careful to abide by Twitter’s terms of service as well as privacy laws [31].

Social media fills a gap by providing real time data about extreme events which is difficult to obtain otherwise. It provides excellent validation data for forecast models as well as live, on-the-ground updates during extreme events. As it stands the method presented in the paper is a proof of concept—social sensing using Twitter can provide accurate and useful information about flooding—and the method presented should already be useful for groups interested in verifying flood forecasts, managing flood responses in real time or tracking the social impact of floods.

## Supporting information

S1 File S1_File.csv: CSV file containing the Met Office Flood Forecasting Centre historical flood data. (CSV)
